# Factors Associated with Asthma ED Visit Rates among Medicaid-enrolled Children: A Structural Equation Modeling Approach

**DOI:** 10.3934/medsci.2017.1.71

**Published:** 2017-02-10

**Authors:** Luceta McRoy, George Rust, Junjun Xu

**Affiliations:** 1 Southern Adventist University, Department of Business, Collegedale, TN 37363; 2 Center for Medicine and Public Health, Florida State University College of Medicine, 1115 W. Call Street, Tallahassee, FL 32304; 3 National Center for Primary Care, Morehouse School of Medicine Atlanta, GA 30310

**Keywords:** Asthma, Local-area variation, emergency department, Medicaid, structural equation modeling, conceptual model, social determinants

## Abstract

**Background:**

Asthma is one of the leading causes of emergency department visits and school absenteeism among school-aged children in the United States, but there is significant local-area variation in emergency department visit rates, as well as significant differences across racial-ethnic groups.

**Analysis:**

We first calculated emergency department (ED) visit rates among Medicaid-enrolled children age 5–12 with asthma using a multi-state dataset. We then performed exploratory factor analysis using over 226 variables to assess whether they clustered around three county-level conceptual factors (socioeconomic status, healthcare capacity, and air quality) thought to be associated with variation in asthma ED visit rates. Measured variables (including ED visit rate as the outcome of interest) were then standardized and tested in a simple conceptual model through confirmatory factor analysis.

**Results:**

County-level (contextual) variables did cluster around factors declared *a priori* in the conceptual model. Structural equation models connecting the ED visit rates to socioeconomic status, air quality, and healthcare system professional capacity factors (consistent with our conceptual framework) converged on a solution and achieved a reasonable goodness of fit on confirmatory factor analysis.

**Conclusion:**

Confirmatory factor analysis offers an approach for quantitatively testing conceptual models of local-area variation and racial disparities in asthma-related emergency department use.

## 1. Introduction

Asthma is one of the most common chronic childhood diseases in the United States, affecting approximately 7 million children [1]. It is one of the leading causes of emergency department (ED) visits and school absenteeism among school-age children [2,3], and is a significant cause of preventable hospitalizations and even death when poorly managed [4].

There is substantial racial-ethnic variation in childhood asthma prevalence, treatment, healthcare utilization, and health outcomes. Each year, approximately two million children under the age of 18 are likely to present to the emergency room for asthma treatment, and disproportionate numbers of these children are from minority groups and low income families [5]. Across racial groups, asthma prevalence is two to three times greater among non-Hispanic black or African American children compared to non-Hispanic whites, and non-Hispanic blacks are also twice as likely to have an asthma-related ED visit compared to other groups [6,7]. Hispanic and Latino children are also more likely to have asthma and to utilize the ED compared to non-Hispanic whites [8]. Within Hispanic and Latino segments of the population, Puerto Rican children have a higher prevalence than those of Mexican descent.

There is also substantial geographic variation in asthma prevalence and health outcomes. Prevalence rates are higher in urban regions than in rural areas, but also vary at the local level [40]. Asthma prevalence and morbidity, as well as racial-ethnic disparities in child asthma emergency department visit rates, can differ across similar or even adjacent communities [9,41].

Measures of socioeconomic status (SES) [10,11], health care systems [12], individual health factors, parental beliefs, and environmental factors such as air quality are all linked to asthma-related health outcomes and utilization. These “determinants” are not only independent risk factors, but are also highly inter-related. For example, race and SES both independently predict asthma prevalence and outcome, but the effects vary by race, with blacks experiencing a higher level of variation [9,13]. Low SES is also associated with limited access to quality healthcare and reduced ability to avoid asthma triggers. With unemployment or lower income, individuals have fewer options for housing, and may live in sub-standard housing with increased exposure to indoor asthma triggers such as mold or cockroach antigen, or to live in neighborhoods with external environment factors that also can increase risk of asthma exacerbations[ 13,14].

Many models have been put forward to explain variation in asthma outcomes, ranging from generic health care utilization models of Anderson and Aday [15] to more recent social-ecologic models promoted by the CDC [16]. EPA has produced variations of these models which emphasize environmental air quality (both indoor and outdoor air quality) [17,18]. Models focused on the individual level emphasize individual self-management behaviors, parental support and knowledge, family dysfunction, etc. However, the interrelationship of conceptual factors has often not been quantitatively tested as comprehensive models.

Over the past decade, structural equation modeling (SEM) has increasingly been used to test conceptual models (using latent factors to represent conceptual constructs) and to address the multifactorial drivers of asthma outcomes, but mostly at an individual level [19,20]. To date, these structural equation models have not been used to understand local area variation in population-based asthma outcomes, nor to understand why racial-ethnic disparities in asthma outcomes vary from one community to another. Therefore, we undertook this study in order to attach measured variables to a simple conceptual model of factors related to variation in outcomes (childhood asthma ED visit rates), and to quantitatively test the goodness of fit of this model (e.g., to explain covariance of all variables in the model related to these conceptual factors). We sought to understand the extent to which real-world data align with our conceptual “mental models” of asthma outcome variation.

## 2. Methods

### 2.1. Study design

**Figure 1:**
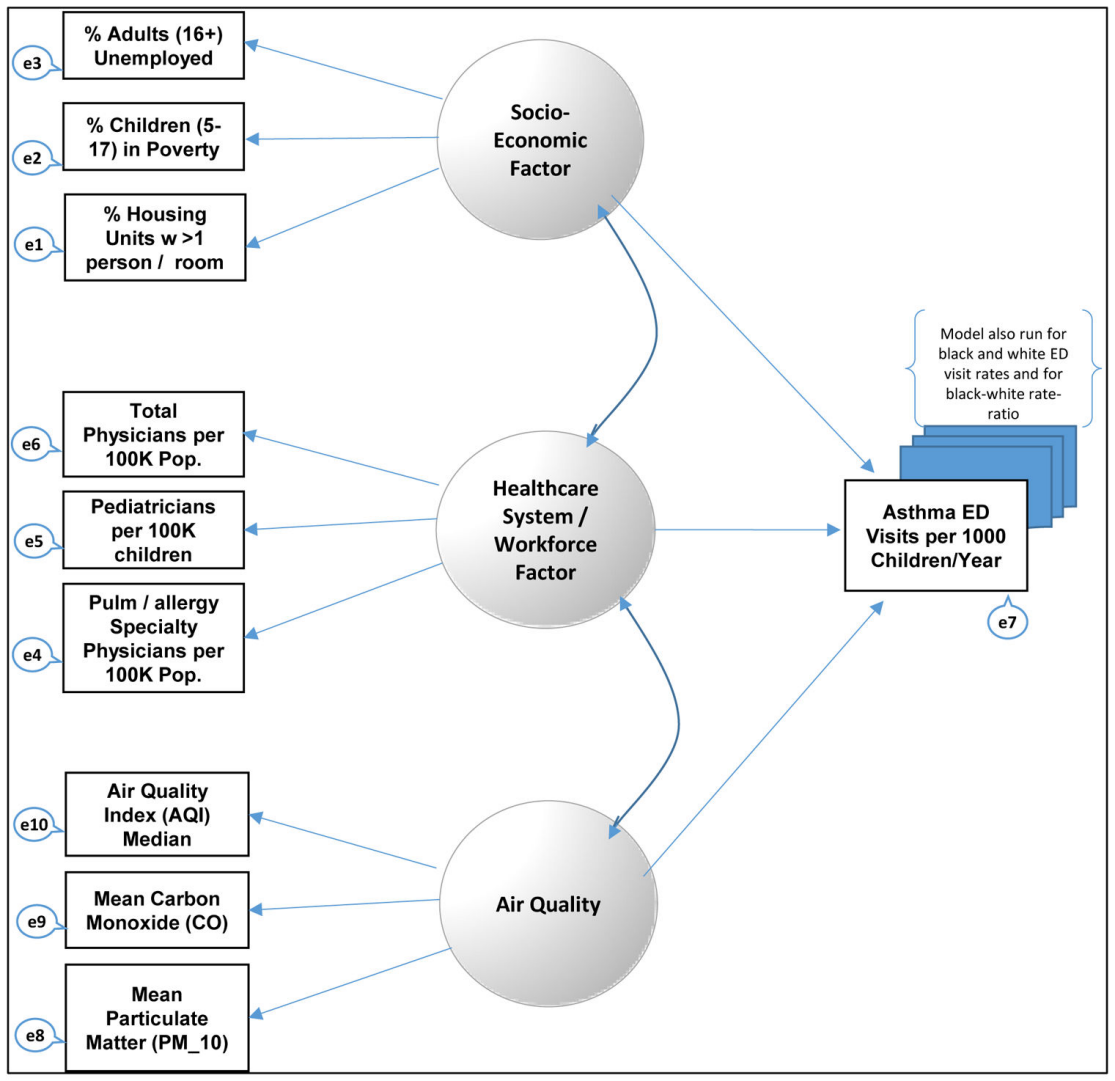
Confirmatory Factor Analysis Model.

We first identified manifest (measured) variables associated with each conceptual construct in a simple conceptual model that we established *a priori.* Then using the SEM approach, we conducted a confirmatory factor analysis (CFA) of manifest (measured) county-level variables associated with factors (latent variables) in the *a priori* conceptual model. Figure 1 shows the relationship between manifest (measured) variables and conceptual factors in the model.

### 2.2. Study population

This study included Medicaid-enrolled children from 28 states (Alabama, Arizona, Arkansas, California, Colorado, Connecticut, Florida, Georgia, Illinois, Indiana, Louisiana, Maryland, Massachusetts, Michigan, Mississippi, Missouri, New Jersey, New Mexico, New York, North Carolina, Ohio, Oklahoma, Pennsylvania, South Carolina, Tennessee, Texas, Virginia, and Washington) and from the District of Columbia. These states represent 80% of all Medicaid enrollees in the country (and 90% of all black or African American and Hispanic or Latino Medicaid enrollees). Medicaid administrative claims data for 2009 were obtained from the Centers for Medicare and Medicaid Services (CMS) in a standard Medicaid Analytic eXtract (MAX) file format. We used data from the personal summary, inpatient, and outpatient files, which contained a 100% sample of enrollees and claims from each state. County level contextual data were acquired from the 2009–2010 Area Health Resource File (AHRF). Twenty two county level air quality measures were obtained from public datasets from the Environmental Protection Agency (EPA) (based on local monitoring sites). We merged the AHRF and the EPA data with the MAX claims data by matching the state-county (Federal Information Processing Standard) FIPS codes.

There were 615,432 children between the ages of 5 and 12 with an asthma diagnosis for at least one inpatient admission or at least two encounters on different dates in the outpatient file, using the International classification of Disease 9 (ICD_9) diagnostic codes 493.xx, excluding 493.2x (Chronic Obstructive Asthma-related to COPD or Bronchitis).

### 2.3. Sample

To protect confidentiality, we included only the counties with at least 11 Black and 11 White children with asthma, which also had air quality data available from EPA public data. The final sample included 265 counties.

## 3. Measures

While the ED visit rates represented the primary outcome measures, they are not dependent variables in a statistical sense, since in CFA the entire model is being tested for its ability to explain covariance of all measures within the model. Our model was tested with several alternative outcome variables measured as the child asthma ED visit rate for all children, then for white NH and black NH children. The model also was tested with disparities as the outcome measure (black-white child asthma ED visit rate-ratio).

County-level independent variables included in the exploratory factor analysis came largely from the Area Health Resources File (AHRF). This AHRF dataset has 6626 variables on each county in the US, covering variables ranging from the health care professional workforce, hospitals and health care facilities, census data, socioeconomic indicators, and environmental measures. Two hundred and twenty-six (226) variables related to health personnel, health facilities, social determinants, and demographic population profiles were considered to be potentially relevant to community-level asthma outcomes. In order to include overall patterns of ED visit use vs. use of physician office visits, we computed rates of all-cause use of the emergency department divided by all-cause physician office visits for each county. To make the diverse scaling of each of those variables more comparable, each variable was standardized and the z-score for each value was used in the confirmatory factor analysis.

These variables were analyzed in exploratory factor analysis (varimax rotation) to test whether or not they clustered into factors that were consistent with our initial conceptual models. Measured variables with higher factor loadings on a single factor were selected for use as manifest variables tied to that factor in confirmatory factor analysis.

## 4. Confirmatory Factor Analysis

County-level socioeconomic status, air quality, and healthcare system capacity were the three conceptual factors (latent variables) in the final models. Several alternative air quality measures relevant to asthma (particulate matter, nitrogen oxides, ozone, median air quality index, and % days of poor air quality) were tested as manifest variables measuring our conceptual construct of “air quality” to find variables that optimized model fit.

Manifest variables were all measured at the county-level, and derived from public data sources as described above. In the confirmatory model, we used as measures of socioeconomic status (SES) the unemployment rate, the percentage of children between ages 5 and 17 in poverty in the year 2008, and the percentage of occupied housing units with more than one person per room between 2006 and 2010. Measures of health system professional capacity include total physicians per 100,000 population, pediatric physicians per 100,000 children in the population, and total pulmonary plus allergy specialists per 100,000 general population.

Asthma outcomes were represented in the model by a single measured variable, which was the county-level asthma ED visit rate per 1000 asthma-diagnosed children (all racial groups). We also re-ran the model with alternative health outcome variables, including the child asthma ED visit rate for white NH children and again for black NH children. The model also was tested with disparities as the outcome measure (black-white child asthma ED visit rate-ratio). The full model of the conceptual variables and their associated manifest variables are shown in Figure 1, and also summarized in Table 1.

Finally, we performed confirmatory factor analysis to assess the goodness of fit of these models, and to understand the extent to which real-world data align with our conceptual “mental models” of asthma outcome variation. The confirmatory approach allowed us to test our *a priori* model and to explicitly define the association between measured variables and factors (latent variables). To assess the fit of the model to our data adequately, we used an absolute measure called the Root Mean Square Error of Approximation (RMSEA), and relative or incremental-fit measures (Normed Fit Index [NFI] and Comparative Fit Index [CFI]) with associated 95% confidence intervals. The RMSEA is deemed a “reasonable” model fit with a value below 0.08 (and an optimal or close fit if the value is below 0.05). CFI and NFI greater than 0.9 were considered to be a satisfactory model fit, while values >0.95 would be optimal [21–23]. The analyses were carried out using SPSS AMOS, version 22.

## 5. Results

Figure 1 shows the configuration of the model. The standardized regression weights for the model are also shown in Table 2. The rate of children age 5–17 in family poverty and unemployment rate, significantly contributed to the latent variable, SES; pediatric physicians per 100 thousand children and total physicians per 100,000 population significantly contributed to the latent variable, health systems and median air quality index significantly contributed to the latent variable, air quality. Of the three latent variables, only health systems had a significant direct association with asthma ED visit rate.

Model fit measures are shown in Table 3. Of the fit indices, only one was not within acceptable range (Chi Square (*Χ*^2^ = 56.819, df = 18, *p* = 0.000), and chi-square is known to be an unreliable measure of fit with larger sample sizes. The CFI and NFI were both above 0.90 at 0.96 and 0.94 respectively. The RMSEA was within the acceptable range (< 0.80) at 0.074, although not optimal (which would be <0.05). Fit indices were also in the reasonable-fit or optimal-fit range for the model stratified for black child ED visits, white child ED visits, and for the black-white rate-ratio of ED visit disparities.

## 6. Discussion

Our findings confirm that asthma ED visit rates are associated with a complex mix of variables, and that our conceptual models of local-area variation in asthma ED visit rates can be quantitatively measured for goodness of fit against real-world data. Our confirmatory factor analysis revealed that the conceptual constructs (latent variables) of socioeconomic status, air quality, and health systems professional capacity were all critical factors in explaining the overall covariance of a model that included asthma ED visit rates as an outcome, whether for all children or for race-specific outcomes. However, since goodness of fit was reasonable by some measures and optimal by others, there is clearly potential for better models (more complex conceptual constructs, different variables, different paths, etc.) to be constructed.

Our work offers a direct methodologic approach to testing various conceptual models of asthma outcome variation and specifically for testing explanatory models of racial disparities in asthma outcomes. It also affirms the multifactorial nature of this variation. While socioeconomic status is important, air quality and health care system factors are also important. Model-testing also gives us a baseline for testing better models and better measures. For example, better measures of healthcare quality or patient behaviors could enhance the model. We also need more timely or granular measure of air quality tied to the time and place of the patient in the days leading up to an ED visit. Similarly, the sustained goodness of fit when we ran the model with a racial disparity outcome measure (racial inequality of ED visit rates as measured by black-white rate-ratio) suggests an opportunity for testing of other disease-specific disparity models or even broader health equity models across myriad diseases, or for testing additional measures more specifically related to socioeconomic or healthcare inequalities (e.g., Gini Index, residential segregation indices, etc.).

Focusing on specific latent variables, our findings support the relationship between socioeconomic status and ED visit rates (both total and race-specific rates). Socioeconomic status has long been implicated as the cause of asthma disparities impacting quality of life, health outcome and healthcare use [24,25]. At the individual level, children whose parents are underemployed or from families with lower income are more likely to have asthma [26,27]. Children whose parents have lower educational status are also more likely to have worse control of asthma [28]. At a local-area level, studies have also linked the risk of asthma to stress from poverty, violence, and neighborhood disadvantage, such as poor housing and overcrowding [29]. Other societal factors are the cultural and social components of the communities in which individuals live, as well as the lifestyles and other factors that may affect the health of the individuals (such as community support and resiliency).

However, social determinants at the individual or neighborhood level do not entirely explain local-area variation in asthma outcomes. Asthma ED visit use can also be explained by access to treatment and quality of care [30]. Our model only represented this factor with variables measuring the strength of the asthma-relevant health professional workforce, but even access to care may be hampered by availability of public transportation to reach health care providers, or low availability of linguistically or culturally competent providers among Hispanic or Latino children [31,32]. Future models will also need to break out quality of care (as measured by under-staging of asthma and clinical inertia or under-prescribing of long-term controller medications), as well as patient adherence to self-management and long-term controller regimens. These are all healthcare factors that affect asthma outcomes [33,34]. Hence racial-ethnic disparities in ED visit rates may reflect the complex interplay of individual and neighborhood-level social determinants, access to care, and quality of care, across differences in sub-group prevalence and severity of illness in minority groups or those of lower income status.

Indoor and outdoor air quality both have been strongly associated with asthma outcomes. Proximity to coal-fired electricity-generating power plants, exposure to farm, dust, tree and grass and other plant allergens all vary geographically and are associated with increased cases of chronic diseases such as asthma [35]. Studies have found significant positive associations between specific components of air quality, including nitrogen oxides, ozone levels, and particulate matter (PM_2.5_ and PM_10_) and ED visits [36]. A study across Ottawa using the Canadian Air Quality Health Index (AHQI), which measures the combined impact of three pollutants (NO_2_, Ozone, and PM_2.5_), found that a one-unit increase in the AHQI was associated with a 5.6% increase in asthma outpatient visits and a 2.1% increase in asthma hospitalizations on the same day of the rise AHQI, and a 1.3% increase in the rate of asthma ED visits after a two-day lag [37]. County-level measures of indoor air quality are more difficult to obtain, but individuals residing in poor residential areas and public housing may have greater exposure to mold and cockroach antigen, dust, poor air filtration, and indoor pesticide use, all of which may be associated with increased risk of childhood asthma exacerbations[ 38].

Our study demonstrated an approach to test our conceptual or explanatory models of asthma outcome variation, but we recognize significant limitations. For example, our conceptual model did not include individual-level predictors of outcomes, such as self-management, trigger-control, and adherence to controller medications. Multi-level structural equation models must be developed to test the complex inter-relationships between individual and county-level contextual factors. Medicaid claims data also provide no information about clinical measures of severity, nor of family dynamics or social supports, nor about the presence or absence of asthma-friendly school health policies, nor a myriad of other factors which could be included in more complex or nuanced models. Future studies will also need more robust air quality data (multiple measures) linked both geographically and temporally to the time and place of the child’s asthma exacerbation.

## 7. Conclusions

The complex interplay of all these factors drives the search for conceptual or explanatory models [39]. To date, explanatory models have rarely been tested rigorously for model fit and validity. Use of SEM multivariate techniques allows for the explicit testing of explanatory models that involves several relationships between observed (measured or manifest) variables and latent variables (conceptual constructs such as poverty or air quality or healthcare system capacity). Future testing of explanatory models can add more robust measures of air quality, test the relationship between individual and contextual variables, and test more complex relationships between the factors (e.g., SES and healthcare system factors as mediators of long-term controller use effect on ED visit rates). Future studies may also demonstrate the application of confirmatory factor analysis as a method to test the overall fit of conceptual models for other conditions with significant racial and local-area variation in chronic disease outcomes.

## Figures and Tables

**Table 1:** Manifest (measured) variables associated with each conceptual (latent) factor.

Conceptual Factor	Measured (Manifest) Variables*
Socioeconomic status (county-level)	% children age 5–17 below poverty % unemployment rate (age >16) % housing units with >1 person per room
Healthcare system capacity (county-level)	Pediatricians per 100,000 children Asthma-related sub-specialists (pulmonary + allergy-immunology) per 100,000 total population Total physicians in practice per 100,000 population
Air quality (county-level)	Mean Carbon Monoxide level Mean Particulate Matter (PM_10_) Median Air Quality Index *(on days measured at EPA monitoring sites in county)*
Asthma outcomes (county-level)	Asthma ED visit rate per 1,000 asthma-diagnosed children *(run as single measured variable; then re-run separately for black and white racial strata and for black-white rate-ratio)*

* Note: All variables have been standardized as z-scores to achieve similar scaling.

**Table 2:** Parameter Estimates from Confirmatory Factor Analysis.

	Unstandardized Parameter Estimates	Standardized Parameter Estimates	C.R.	*p*-value
***Factor = Socioeconomic (SES) Indicators***				
• Occupied Housing Units with >1 Person per room	1.000	0.574		
• Unemployment Rate (age >16)	0.582	0.454	4.403	<0.001
• Related Children 5–17 in Family Poverty	0.487	0.317	3.401	<0.001
***Factor = Health Systems/Workforce***				
• Total Pulmonology Specialty Physicians per 100K population	1.000	0.898		
• Pediatric Physicians per 100K children	0.867	0.926	23.460	<0.001
• Total Physiciansper 100K population	1.046	0.999	28.254	<0.001
***Factor = Air Quality***				
• Mean Particulate Matter (PM_10_)	1.000	0.652		
• Median Air Quality Index (AQI)	1.422	0.853	4.163	<0.001
• Mean Carbon Monoxide level	0.217	0.145	1.154	0.248

**Table 3:** Comparison of Goodness of Fit Tests for Confirmatory Models with 3 Different Outcome Measures with and without Air Quality as Variable in Model.

	Asthma ED Visits: All Children	Asthma ED Visits: Black Children	Asthma ED Visits: White Children	Black-White ED Visit Rate-Ratio
Air Quality Included as Variable in Model				
**RMSEA**	0.098	0.095	0.097	0.100
**CFI**	0.956	0.958	0.956	0.953
**NFI**	0.938	0.940	0.938	0.935
Air Quality ***Not***Included as Variable in Model				
**RMSEA**	0.080	0.074	0.078	0.084
**CFI**	0.980	0.983	0.981	0.978
**NFI**	0.967	0.970	0.967	0.965
